# Outcomes and evaluation of a National Institutes of Health funded training program for doctoral students: The Jackson Heart Study Graduate Education and Training Center at the University of Mississippi Medical Center

**DOI:** 10.1016/j.evalprogplan.2025.102606

**Published:** 2025-05-10

**Authors:** Olivia Affuso, Lecretia Buckley, Abigail Gamble, Elizabeth Heitman, Rachel S. Tyrone, Jennifer C. Reneker

**Affiliations:** a Council on Black Health, Knightdale, NC, USA; b Department of Advanced Biomedical Education, School of Medicine, University of Mississippi Medical Center, Jackson, MS, USA; c Department of Preventive Medicine, John D. Bower School of Population Health, University of Mississippi Medical Center, Jackson, MS, USA; d Myrlie Evers-Williams Institute for the Elimination of Health Disparities, University of Mississippi Medical Center, Jackson, MS, USA; e Program in Ethics in Science and Medicine, University of Texas Southwestern Medical Center, Dallas, TX, USA; f Department of Population Health Science, University of Mississippi Medical Center, Jackson, MS, USA; g Department of Otolaryngology and Head and Neck Cancer, University of Mississippi Medical Center, Jackson, MS, USA; h Department of Research and Sponsored Programs, Northeast Ohio Medical University, Rootstown, OH, USA

**Keywords:** Doctoral training, Research mentoring, Program evaluation, Cardiovascular epidemiology, Biomedical sciences

## Abstract

**Introduction:**

A novel Graduate Training and Education Center (GTEC) under the National Institutes of Health funded Jackson Heart Study (JHS) at the University of Mississippi Medical Center (UMMC) was launched for doctoral students from backgrounds underrepresented in biomedical sciences. UMMC GTEC supports scholars through a program in cardiovascular epidemiology with research training institutes, scientific mentoring, and participation in professional coaching. This manuscript describes the program’s origins, features, and evaluation findings, and discusses the feasibility and limits of complementary research training and mentoring to enhance the biomedical research workforce.

**Methods:**

A program evaluation framework was used to describe the processes, outcomes, and lessons learned. Data from program graduates were synthesized using a convergent parallel design.

**Results:**

Between 2021 – 2024, 22 scholars graduated from UMMC GTEC, all of whom were from groups characterized by the National Science Foundation as underrepresented in biomedical research in the United States. There was evidence of convergence between the qualitative themes with eight quantitative measures to support the findings of significant increases in self-efficacy for science communication, varying changes in career outcome expectations, and no significant changes in science identity. Five findings diverged on career interests and mentor influence. Five scholars (22.7 %) submitted their UMMC GTEC project manuscripts to a peer-reviewed journal, four (80.0 %) of which have been published. Fifteen of 22 scholars (68.2 %) submitted abstracts for presentation at national conferences.

**Discussion:**

The feasibility of peer-reviewed publication during the training program was low but the submission of conference abstracts from completed projects was high. Mentors worked effectively with each scholar, and the program contributed positively to the number of next generation cardiovascular epidemiology researchers in Mississippi.

## 1. Introduction

The Jackson Heart Study (JHS) is a 25-year-old longitudinal cohort investigation of genetic, behavioral, and environmental risk factors associated with the disproportionate burden of cardiovascular disease (CVD) among African Americans, funded by the National Institutes of Health’s (NIH) National Heart, Lung, and Blood Institute (NHLBI). The primary aim of the JHS is to advance understanding of cardiovascular health through epidemiologic research and community engagement. The initial JHS cohort included over 5000 African American residents of the three-county Jackson, Mississippi metropolitan area (Howard & Howard, 2020), some 3000 of whom are still active participants.

**Fig. 1.**
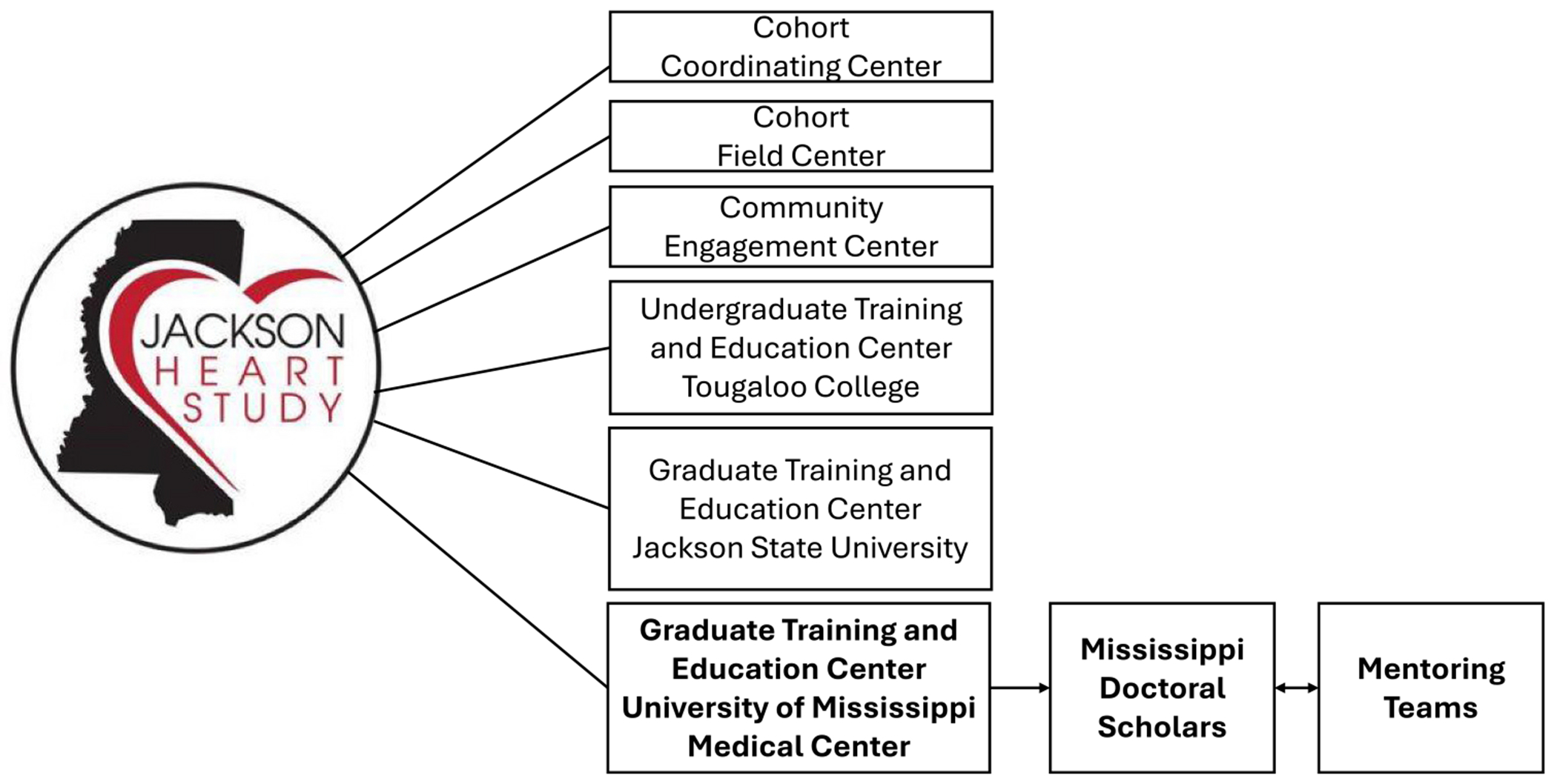
Organizational chart of the Jackson Heart Study.

A second, equally important aim of the JHS is to cultivate the next generation of biomedical scientists and enhance the expertise of Mississippi’s biomedical research and public health workforce. To this end, NHLBI supports three JHS training and education centers: Tougaloo College has the Undergraduate Training and Education Center (White et al., 2023; Srinivasan, 2005), and Jackson State University (Addison, 2021), and the University of Mississippi Medical Center each have a Graduate Training and Education Center (Fig. 1). These programs were funded in response to NHLBI’s request for proposals (NOT-HL-17–544) and designed to provide undergraduate, master’s-level, and doctoral-level scholars, respectively, with supplemental skills training and mentored research opportunities that support the development of their understanding of cardiovascular diseases and epidemiologic research (National Institutes of Health US, 2017).

The University of Mississippi Medical Center Graduate Training and Education Center (UMMC GTEC) was launched in 2018 by a multidisciplinary team of experienced researcher-educators. Its two-year program for doctoral students was designed based on the Social Cognitive Theory (Byars-Winston et al., 2011) to integrate research training, mentoring, and career coaching to support scholars from backgrounds underrepresented in biomedical science through intensive research training institutes, regular individualized research mentoring meetings, professional coaching, participation in national scientific meetings, and production of peer-reviewed scientific publications utilizing JHS data. This type of workforce training program is novel in its connection to an NIH-sponsored epidemiologic study and in the complementary, rather than primary, nature of the training it provides.

## 2. Background and state of research

For over three decades, the biomedical research enterprise in the United States has been characterized by mounting shortages of skilled investigators able to meet the nation’s demand for basic, clinical, translational, and population health research. Multiple national reports have documented the significant need to expand the biomedical research workforce and the complexity of the situation that is increasingly considered a crisis (Daniels & Beninson, 2019; Garrison & Ley, 2022; Gromova & Grieco, 2024; Institute of Medicine US, 2003; National Research Council US and Institute of Medicine US, 2006; National Academies of Sciences, 2018). One component of this growing crisis is the additionally persistent underrepresentation of racial and ethnic minority populations in the biomedical workforce, a phenomenon that is also linked to health disparities across the nation (National Academies of Sciences, Engineering, and Medicine, 2024; Institute of Medicine US Committee on, 2003).

Since the 1990s, the National Institutes of Health (NIH) has implemented various strategies to expand the number of young investigators from underrepresented backgrounds in biomedical research. The NIH has funded new initiatives for workforce development, sought and promoted innovative models of graduate and post-doctoral research training to increase the recruitment and retention of trainees from underrepresented backgrounds, and in 2014 established the Office for Workforce Diversity (Valantine, Lund, & Gammie, 2016; National Research Council US, 2005; National Institutes of Health US, 2018; NIH (US)). In 2017, to improve population health and workforce diversity in Mississippi, NHLBI sought proposals for two “Training and Education Centers for cardiovascular epidemiology and related biomedical research, targeting underrepresented minority undergraduate and or post-graduate students and leveraging the resources and research opportunities of the JHS”. Consistent with the NHLBI’s detailed RFP and requirements to implement a training center in Mississippi for learners in Mississippi colleges and universities, the technical objectives of UMMC GTEC are to:

1. Provide intensive epidemiology research training that includes hands-on research experience, guidance in manuscript writing and scientific presentations, senior and near-peer mentors, and career navigation.
2. Strengthen participating scholars’ knowledge of scientific research by establishing a graduate-level program focused on cardiovascular epidemiology in the context of improving the health of Mississippians.
3. Produce and disseminate research publications and informational articles on educational topics for broader audiences; present research at professional conferences; and participate as members of the scientific community.
4. Encourage students to pursue further training for future biomedical research careers.

This paper describes the unique features of UMMC GTEC, examines the program evaluation outcomes for four completed cohorts, discusses the feasibility of concurrent research training and mentoring for doctoral students, and provides an overview of how UMMC GTEC addressed the program goals established by NHLBI and UMMC GTEC faculty.

## 3. Methods: program overview and implementation

The UMMC GTEC is a two-year, evidence-based complementary epidemiology research training and mentoring program for doctoral students, designed to run concurrently with and in addition to scholars’ primary doctoral programs in other biomedical disciplines. The novelty of UMMC GTEC lies in its provision of education and research activities in addition to the trainees’ education and research activities in their original degree program, along with the enhanced research mentorship from faculty not affiliated with their home university. In this approach, UMMC GTEC aims to prepare the next generation of biomedical scientists and foster their research careers in biomedical sciences through a curriculum that focuses on enhancing knowledge and skills in population-based cardiovascular epidemiology, cardiovascular health, and career development.

### 3.1. The UMMC GTEC scholars

Annually since 2019, students have been recruited from five institutions in Mississippi that offer health-related doctoral degree programs, but which do not offer graduate degrees in epidemiology. A cohort of up to eight scholars was chosen each spring through a competitive application and selection process. Those who enrolled committed to participation in the activities of the two-year program and received a stipend of $7500/year and paid travel related to UMMC GTEC activities.

### 3.2. Research mentors and career coaches

The UMMC GTEC research mentor team includes faculty from UMMC and other universities nationwide with expertise in epidemiology, minority health, health disparities, research ethics, and data science. The team also has members with expertise in evidence-based research training, adult education, and coaching of doctoral-level learners. The original faculty team organically came together to develop GTEC out of a commitment to mentoring students from underrepresented backgrounds in biomedical research and prior experience with the JHS and cardiovascular health research in Mississippi. Since that time, the faculty team has evolved, with openings filled as necessary, to meet the needs of the contract and ensure educational and mentoring support to the scholars.

In addition to the research mentors, two career coaches work with the scholars. These individuals have expertise in the professional development of graduate students and early career faculty, human resources, career planning, personal and professional goal setting, and coaching. The career coaches work with all the scholars in group training sessions and individual meetings.

### 3.3. UMMC GTEC curriculum

The UMMC GTEC’s formal curriculum incorporates group-based didactics and individual instruction integrated with mentored experience in research design, data analysis, and manuscript preparation. As in other NIH research training grants, UMMC GTEC’s curricular format is structured around two cohort-based, multi-day, in-person Summer Institutes and two shorter Mid-year Meetings, supplemented by reading and short writing assignments through the online course platform (Canvas) and additional webinars on select topics. The UMMC GTEC’s overall curriculum is focused on ten essential components of research:

- Knowledge of fundamental factors in cardiovascular health and disease
- Familiarity with the literature in a selected problem area of cardiovascular research
- Skills in critical reading, assessment of peer-reviewed literature, and identification of gaps in evidence and new research questions
- Conceptual understanding and practical skills in study design, data management, and analysis of secondary data
- Scientific writing of proposals, abstracts, posters, and manuscripts
- Oral communication of research findings and use of presentation software
- Standards, policies, and challenges in responsible conduct of research (RCR)
- Interdisciplinary research collaboration and team communication
- Professionalism and time management
- Project management and leadership

Through formal and informal program review and feedback from the faculty and scholars, the UMMC GTEC program has evolved and has been continuously improved. The description provided below is directly reflective of the experience for the most recent cohort of scholar graduates (2024).

### 3.4. Summer institute I (Year 1)

In the first summer, scholars attended the four-day Summer Institute I. Didactic sessions concentrated on foundational knowledge of cardiovascular epidemiology, principles of minority health, and health disparities research as exemplified in the goals, history, structure, and contributions of the JHS, together with the role and objectives of the NIH and NHLBI. This conceptual framework was provided in tandem with practical instruction on defining a research question and developing an appropriate study design using existing JHS data; approaches to statistical analysis and introduction to statistical programs; strategies in searching for, identifying, and critically reading relevant literature; and basics of scientific writing and publication, including standards of responsible authorship and citation.

Together with this emphasis on concepts and tools for epidemiologic research, students received focused instruction in RCR, including the professional norms and culture of academic research, insights into the research mission and social responsibilities of the university, the challenges of objectivity and bias, formal policies and professional standards of research integrity, and the goals and expectations of mentor-trainee interaction. These sessions formed the basis of UMMC GTEC’s RCR curriculum and were reflected across the curriculum with group discussion and advising sessions.

Formal didactic sessions were complemented by individualized mentoring sessions on developing a research question, consultations with UMMC GTEC’s reference librarian on conducting effective literature searches and reference management, and career planning, including creating an individual development plan and individual SWOT analysis with the program’s career coaches.

At the end of the Summer Institute I, as scholars identified their research areas and personal goals for the program, each was paired with a primary mentor with parallel or complementary interests able to supervise and promote the scholars’ work. The program director then established a three-person mentoring team for each scholar that included an epidemiologist and a biostatistician.

### 3.5. Experiential learning: literature review and initial question development

The experiential learning component of UMMC GTEC was each scholar’s mentored development of a research manuscript proposal using JHS data, the presentation of their completed research project at a national conference, and the publication of their manuscript in a peer-reviewed journal.

The first task in preparing a manuscript proposal was defining the research question to be answered using JHS data. For this assignment, scholars read the JHS methods and background papers (Taylor, 2005; Taylor, 2005; Payne, 2005; Dubbert et al., 2005; Keku, 2005), explored the JHS website and the online variable finder JHS (2024), reviewed additional JHS research publications relevant to their area of interest, and examined previously approved JHS manuscript proposals. Scholars completed these initial tasks largely independently, consulting with their mentors as needed. Once they identified their research question, scholars gathered relevant literature and scheduled a virtual meeting with their mentoring team. At this meeting, scholars gave their first work-in-progress presentation, allowing the mentors to ask questions and assist them in further refinement.

At this stage, scholars began to write their formal *manuscript proposal*, a structured document that must be approved by the JHS Publications and Presentations Subcommittee (PPS) before JHS data can be shared with any researcher. Through a collaborative and iterative process, each scholar wrote their proposal, submitted it to their mentors, received written and oral feedback, revised and resubmitted until the proposal met the mentors’ standards and was ready to be submitted for PPS review.

### 3.6. Mid-year meeting I

Between the Fall and Spring semesters, UMMC GTEC scholars and mentors came together again in person for individual and group sessions during the three-day Mid-Year Meeting I. This meeting was designed to strengthen scholars’ respective projects and deepen their statistical knowledge and skills in data analysis. Didactic sessions focused on biostatistical analysis of epidemiologic data, tracking relevant literature, and using statistical and reference management software. The meeting also served as a protected time for scholars to write, refine, and present their manuscript proposals to the group, with peer review provided by their colleagues and mentors. Group-based academic career coaching focused on developing an academic curriculum vitae, an NIH biosketch, and an NCBI My Bibliography page. Individual sessions with the career coaches, reference librarian, and mentoring teams allowed scholars to tailor instruction and guidance to their disciplinary goals and needs.

Didactic RCR sessions at the first Mid-Year Meeting focused on standards of data management, ownership, and sharing, and introduction to institutional structure for regulatory and ethical approvals (e.g., IRB protocol review and Data and Materials Distribution Agreements [DMDAs]). These sessions highlighted federal requirements and the individual institution’s role in interpreting federal policy and providing ethical and regulatory oversight of research. These sessions prepared scholars for the upcoming practical experience of seeking IRB approval and navigating the institutional processes for completing an institutional DMDA with the JHS Coordinating Center.

At the end of the first Mid-Year Meeting, scholars typically had a clear research question and initial hypothesis, relevant supporting literature, an idea for how to present the logic defining the gap in the literature, a preliminary list of variables from the JHS, a preliminary statistical analysis plan, and a schedule for the subsequent mentoring meetings needed to complete and submit the manuscript proposal by mid-Spring.

### 3.7. Experiential learning: completing the manuscript proposal for submission

After Mid-Year Meeting I, the collaborative, iterative writing process between each scholar and their mentoring team continued. The scholars also continued to locate, read, and incorporate background literature to refine their study questions and build their individual knowledge base. By mid-to-late Spring, most scholars had a manuscript proposal formally approved by each member of their mentoring team, some with additional contributions from an identified external expert in the topic area and were ready to submit it for review by the JHS PPS.

### 3.8. Summer institute II (Year 2)

In their second summer, scholars attended the three-day Summer Institute II, where the focus shifted to the conceptual and practical knowledge needed to translate their individual projects into conference presentations and peer-reviewed publications as deliverables. Upon arrival at this second institute, the scholars presented their works-in-progress to their colleagues, the members of the new cohort of first-year scholars, and the entire mentoring team. Scholars met with career coaches individually to review and reevaluate their IDPs and curriculum vitae.

Summer Institute II primarily focused on developing statistical analysis skills in an R-Studio workshop. Although most scholars did not yet have the JHS data for their individual projects, the workshop used other available datasets to allow scholars to practice statistical techniques so that when they received their data, they would be ready to begin the analysis per their individual project specifications.

In addition to learning more about statistical analytic techniques, scholars participated in didactic RCR sessions focused on recognizing and mitigating bias and conflicts of interest and standards of responsible authorship and publication. Finally, scholars took part in individual in-person mentor meetings to identify a professional conference to which to submit an abstract for presentation in the coming year and identified potential journals for submission of the project manuscript, as well as to plan the timeline for completing their research project.

### 3.9. Experiential learning: completing the institutional review board protocol, data and materials distribution agreement, and analysis of JHS data

Once the manuscript proposal was approved by the JHS PPS, the scholar completed a series of regulatory steps (specific to their respective university) to obtain the data related to their project from the JHS Coordinating Center. This process included navigating institutional structures for regulatory and ethical approvals (e.g., IRB protocol and DMDA) and professional correspondence with institutional officials for signatures. It typically took 2 – 4 months for the required institutional approvals to be completed and for the data to be released by the JHS.

Once scholars received their requested data, they began working with a biostatistical mentor to complete the planned analysis. Depending on the scholar, this analysis was carried out during a screen-shared web-based conference call, through in-person step-by-step tutoring, or by the scholar independently working on the analysis and sharing the code for mentor assurances and oversight (with corrections as necessary). As the data were analyzed, scholars were mentored through writing a conference abstract with their full mentoring team. Before submission of the abstract for consideration for a conference, the JHS PPS reviewed and approved the text and content.

### 3.10. Midyear meeting II

The second three-day Mid-Year Meeting was again held between the end of the Fall and the beginning of the Spring semester of their second year of UMMC GTEC. Depending on where the scholars were with the completion of their data analysis, this meeting focused on completing work on their respective projects. All the mentors attended, and most of the scheduled meeting time was devoted to working meetings, focused discussions and problem-solving, collaborative writing and editing, statistical analysis, and interpretation of results. Depending on the scholar and the speed of progress with their respective project, some scholars worked on developing or refining their conference abstract while others prepared platform or poster presentations for conference submissions already accepted.

Didactic content was limited at the second Mid-Year Meeting, except for RCR sessions on maximizing the value of attendance at professional conferences, professional socialization and networking, and more detailed instruction on developing and delivering oral platform presentations with PowerPoint slides and designing and delivering accurate and appealing poster presentations. Career coaches and scholars also discussed time management strategies, and the development of a research program as opposed to focusing on individual research projects. This session also highlighted academic expectations for faculty appointments, promotion, and tenure.

### 3.11. Experiential learning: collaborative writing and scientific professionalization

After the second Mid-Year Meeting, scholars continued to meet with their mentoring teams as needed and collaborated on the iterative writing process. For scholars who were preparing for professional presentations at conferences, the mentoring team hosted a series of web-based sessions in which scholars practiced delivering their presentations and received feedback on the content and delivery of their slides or poster content, with the goal of the scholar being comfortable and confident with their presentation and ready to present. The final product of the manuscript proposal was the completed manuscript. Before submission to a journal for peer review, a completed manuscript must again be reviewed and approved by the JHS PPS.

### 3.12. Celebration of scholarship and graduation

In the Spring, after the second Mid-Year Meeting, the graduating scholars, first-year scholars, and all the mentors came together for a celebration of scholarship and graduation ceremony. The venue has changed each year, and the event has occurred in concert with the UMMC School of Population Health Research Day, the regional conference of the Mississippi Academy of Science, and the national American Heart Association – Epi Lifestyle Scientific Sessions. This capstone event included a keynote speaker, and each graduating scholar presents their UMMC GTEC project to an expanded audience.

### 3.13. Peer and near-peer interaction

Since 2022, the year 1 and year 2 cohorts’ Summer Institutes and Mid-Year Meetings have been held concurrently to promote peer interaction and encourage collegial development and networking across institutions, departments, and developmental stages within the progression of doctoral training. The UMMC GTEC scholars in their second year have multiple opportunities to present their works in progress; during the Mid-Year Meetings, the first-year scholars are also offered this opportunity. This practice both creates a safe environment for the presenting scholars to receive peer feedback before scientific conferences and for the listening scholars to learn to provide feedback and offers a practical example of peer review and multidisciplinary engagement.

Also, as successive cohorts have completed the program, the past two years have allowed scholars from the early cohorts to return to UMMC GTEC meetings and gather with current scholars at professional conferences. These former scholars completed UMMC GTEC up to 4 years before the active cohorts and are in post-doctoral training and pursuing or entering new faculty positions. This near-peer interaction has encouraged newer scholars’ curiosity about academic life and facilitated personal connections, enabling scholars to learn about early-career faculty investigators’ experiences. Although the outcomes of these interactions have not been formally evaluated, these interactions have resulted in ongoing connections with the establishment of collegial collaborations and friendships (Hayes & Jones, 2024).

### 3.14. Diversity supplement opportunities

In addition to sponsoring the scholars’ individual projects, the UMMC GTEC principal investigator is eligible to apply for NHLBI’s Administrative Supplements, which are intended to increase diversity in the research workforce by providing additional research training opportunities through small projects linked to the parent contract. These “Diversity Supplements” are encouraged as part of UMMC GTECs’ Task Orders to expand the program’s impact NIH (US). Since its inception, UMMC GTEC has applied for and received funding for two Diversity Supplements, one for a postdoctoral scholar and one for a pre-college scholar. Their funded research resulted in one poster presentation at a national conference and one peer-reviewed publication (Reeder & Reneker, 2024).

### 3.15. Program impact evaluation

A convergent parallel design using quantitative pre/post questionnaires and qualitative exit interviews was used to evaluate UMMC GTEC from the perspectives of the scholars. Convergent parallel designs are particularly useful for understanding participants’ experiences and enhancing the validity of program evaluation outcomes (Creswell & Concise, 2015). The quantitative questionnaire was adapted from surveys validated and previously published (Anderson et al., 2016; Chemers et al., 2011). Per the original instruments, each question utilized a 5-point Likert scale, ranging from “very insecure” or “strongly disagree,” scored as 1 to “very secure” or “strongly agree,” scored as 5, to assess changes in mentor influence, scientific communication self-efficacy and interests, career outcome expectations, and identity as a scientist (Anderson et al., 2016; Chemers et al., 2011). The operationalization of each measure is provided in Supplement 1 (S1 Table), using the constructs defined by the original publications (Anderson et al., 2016; Chemers et al., 2011). The supporting qualitative data for each of the themes is provided in Supplement 2 (S2 Table). Assessments were conducted at baseline and end-of-program. Scholars also participated in a brief exit interview using a semi-structured guide to gather feedback on their overall experiences. The interviews were conducted virtually, audio-recorded, and transcribed into Microsoft Word. The program evaluation was granted an exemption from regulatory oversight by the UMMC IRB.

Analysis was conducted in two phases. In phase 1, data sources were analyzed independently. Quantitative analysis was conducted in IBM SPSS Statistics Version 29.0.2.0 (IBM), using a paired, 1-tailed *t*-test to assess mean differences in baseline and end-of-program measures. Significance was established *a priori* at p < 0.05. Two members of the project team coded the interview transcripts using a thematic analysis based on the theoretical constructs measured in the quantitative survey. Data integration was conducted in phase 2 and reported in a side-by-side joint display, including quantitative and qualitative data in parallel and a final column indicating whether the data converged or diverged.

## 4. Results: program outcomes

### 4.1. The scholars

Since its inception in 2019, UMMC GTEC has accepted 38 scholars into the program (S3 Table), 22 of whom have been trained in four cohorts and graduated from the 2-year program. Over this period, four scholars left the program due to scheduling conflicts with their primary program, employment, or health considerations. Currently, 12 scholars are participating in the program, with 6 in Cohort 5, which began in June 2023, and 6 in Cohort 6, which began in June 2024. Across all cohorts, most scholars have been from UMMC (n = 17), followed by the University of Mississippi (n = 12), Mississippi State University (n = 7), and the University of Southern Mississippi (n = 2).

In keeping with the NHLBI’s strategic vision to enable and develop a diverse biomedical workforce and UMMC GTEC’s goal of supporting the development of researchers from groups underrepresented in biomedical science (Lauer et al, 2015; National Institutes of Health US, 2018), all the scholars enrolled across the six cohorts self-identified as being from groups characterized by the National Science Foundation as underrepresented in U.S. biomedical research (National Science Foundation. US, 2023). Of the 38 scholars, 20 (79 %) identified as members of one or more underrepresented racial or ethnic minority groups, 5 (13 %) reported living with a physical or mental disability, and 13 (34 %) reported coming from economically disadvantaged backgrounds.

The scholars’ primary doctoral programs included a variety of disciplines, with 17 (45 %) in clinical programs (i.e., kinesiology, medicine, or pharmacy), 11 (29 %) in basic science (i.e., food science, medicinal chemistry, neuroscience, genetics, etc.), 8 (21 %) in population health or biostatistics, and 2 (5 %) in social work and psychology.

### 4.2. Program impact evaluation outcomes

Complete quantitative and qualitative data were available from 20 scholars representing cohorts 1 through 4. Qualitative analysis yielded five themes that best represented the most salient data (Table 1).

The results of the convergent parallel analysis are reported in Table 2. Overall, data converged on vicarious learning through and affective response to mentors, self-efficacy for scientific communications, career outcome expectations, and science identity, and diverged on mentor social influence and career interests.

There were no significant quantitative changes in mentor influence. Non-significant changes in social influence (mean difference [MD]=−0.40, p = 0.074; negative outcome) and affective response (MD=−0.13, p = 0.310; positive outcome) were noted; however, the standard deviation of each mean difference was greater than 1, suggesting divergent changes in these constructs across participants. The decrease in social influence was contrary to the qualitative data, which showed (Theme 1) *Empathetic and accessible mentorship fosters positive research experiences among doctoral and graduate students*. Significant increases in scholars’ self-efficacy for scientific writing (MD=0.46, p = 0.001), oral presentations (MD=0.62, p = 0.001), and conversations (MD=0.64, p < 0.001) were supported by the feedback provided by mentors, which enhanced confidence in research conduct and scientific communications (Theme 2).

Significant changes in positive career outcome expectations (MD=−0.30, p = 0.003; positive outcome) and no change in negative career outcome expectations (MD=0.21, p = 0.110) were supported by Theme 3. Although not significant, the slight increase in negative career outcome expectations was in the direction of a favorable (positive) outcome. Over the course of the program scholars realized that research is a rigorous and lengthy process, yet many expressed a continued interest in pursuing a career that included research. This finding from the qualitative data (Theme 4) diverged with all measures of career interests including scientific writing (MD=−0.63, p = 0.002), oral presentations (MD=−0.48, p=0.004), and scientific conversations (MD=−0.31, p = 0.046). A final theme emerged – *Science identity emerges through socialization into the norms and discourse practices of science* – which converged with the non-significant upward trend in scientific identity (MD=0.33, p = 0.147).

### 4.3. Scholarly products

Across the four completed cohorts, it has taken an average of ten months (range 4 – 20 months) from Summer Institute I until the manuscript proposal was submitted to JHS PPS for review. In the JHS PPS review process, manuscript proposals are either accepted with comments or returned with requested revisions. On average, receiving final approval has taken 1.25 months from the date of submission to JHS PPS (range: 0.5 – 3 months). All 22 UMMC GTEC graduates successfully prepared a manuscript proposal and received JHS PPS approval. Of these graduates, 15 (68.2 %) have completed and submitted abstracts for oral or poster presentations at professional conferences. On average, it has taken 5.8 months (range 0.5 – 8 months) after JHS PPS approval of a manuscript proposal for scholars to develop and submit their abstract for JHS PPS review and an additional three weeks for approval (range 0.5 – 2 months). Five of the 22 graduates (22.7 %) have submitted completed manuscripts to peer-reviewed journals. On average, it has taken 14.3 months (range 10 – 24 months) after JHS PPS approves the proposal for the manuscripts to be completed and an additional 3.5 months for JHS PPS to approve the completed manuscript (range 1.5 – 10 months).

At this publication’s writing, four of the five (80.0 %) manuscripts first authored by scholars have been accepted for publication (Reeder & Reneker, 2024; Jones, 2021; Booker, 2022; Hayes, 2025), and one is in review. With scholars as co-authors, the mentoring team published two additional manuscripts using data from the JHS Kids Study (Beech, 2021; Bruce, 2021). As of this writing, the fifteen scholar-led abstracts have been accepted and presented at professional conferences, some of which have also been published in peer-reviewed journals (Hayes, 2024; McLaurin et al., 2024; Nasruddin et al., 2023; Patrick et al., 2024; Reeder, 2022; Cohen-Winans, 2023; Jackson et al., 2023). Scholars have presented UMMC GTEC research as platform and poster presentations at the annual meetings of the American Heart Association Epi Lifestyles meeting, the Interdisciplinary Association of Population Health Science, the American Association for the Advancement of Science, the Society of Nutrition Education and Behavior, the Mississippi Academy of Science, and the Mississippi Public Health Association.

### 4.4. Pursuit of further biomedical training

Of the 22 UMMC GTEC graduates, 12 (54.5 %) have completed their doctoral programs; of those, five (41.6 %) have continued their training with post-doctoral positions, and two (9.0 %) have continued into medical residency programs with a research component. This represents 58.3 % (n = 7) of the eligible UMMC GTEC graduates who have pursued further training for future biomedical careers. The other five scholars who have completed their graduate doctoral programs have secured jobs in academic or industry settings or have clinical positions. The remaining 10 UMMC GTEC graduates are working towards completing their doctoral degrees.

## 5. Discussion

Research training programs such as UMMC GTEC are a key component of the NIH’s strategy to promote a diverse scientific workforce and have been shown to increase the representation of scholars from minority populations in science, technology, engineering, and mathematics (Bianchini, 2013). Unlike other cardiovascular epidemiology education and mentoring programs, such as the NHLBI’s PRIDE (Programs to Increase Diversity among Individuals Engaged in Health Related Research) for early career faculty, UMMC GTEC engages scholars earlier in their graduate careers to create a pathway to population-based cardiovascular disease research that may not have otherwise existed (Boyington, 2016).

Three areas of focus are central to a training programs’ ability to equip students for the biomedical research workforce: 1) knowing what is happening within the training pipeline, rather than only counting those entering and those leaving it; 2) supporting and encouraging non-traditional students; and 3) defining and utilizing best practices for academic success (McGee, 2016). The design of UMMC GTEC aligned with the overall framework set forth by the Training and Education to Advance Minority Scholars in Science (TEAM-Science) through mentorship, competency-based training, career coaching, career development, and identifying personal career analysis (Byars-Winston et al., 2011).

UMMC GTEC met its first objective, providing intensive hands-on research experience and manuscript writing through strategic and purposeful research mentoring, guidance, and collaborative writing in which dedicated faculty foster student-driven research projects using JHS data. The program’s design provided scholars with opportunities to develop and disseminate scholarly publications in peer-reviewed journals and deliver presentations at professional conferences. UMMC GTEC provided professional socialization in a community of senior, near-peer colleagues across the curriculum, and structured training activities. Career coaches guided scholars as they navigated future opportunities and career options.

Results from the program evaluation demonstrated improvement in scholars’ self-efficacy related to scientific writing, oral presentations, and conversations, while simultaneously revealing their appreciation for the challenges in these pursuits. The observed quantitative decrease in career interest may be accounted for by the reality that research is a lengthy and rigorous process, which was salient in the qualitative data. Because none of the scholars had prior experience obtaining data for secondary analysis and the protracted processes involved (i.e., manuscript proposal development, submission, and approval; IRB review; and DMDA execution), it is possible that some of the negative findings reflect aspects inherent to research with this type of data.

While there were non-significant changes in mentor influence, the negative shift (albeit small) in mentor social persuasion and the non-significant improvements in vicarious learning and affective response were surprising. The positive interview responses suggested that continuous mentorship increased mentees’ engagement in the extensive research process and emphasized that scholars recognized key skills and professional development as critical components of their future success. Therefore, significant positive quantitative findings were anticipated. One consideration for our results is that the baseline quantitative survey was completed prior to the scholars’ initial interaction with the mentors in the GTEC program, so their baseline responses likely reflected their relationships and interactions with their prior mentors, including their primary program mentor. The end-of-program quantitative questionnaire did not specify that scholars should only consider their interaction and experiences in GTEC when answering the questions, so we do not know the degree to which the answers reflect GTEC only or their combined experiences in GTEC and their primary doctoral program. The qualitative interviews, however, asked questions that were framed as being specific to their GTEC experience. This may explain some of the divergence in some of the findings between the quantitative and qualitative findings.

The UMMC GTEC’s approach to its second objective, to strengthen the scholars’ knowledge of scientific research focused on cardiovascular epidemiology in the context of improving the health of Mississippians, evolved since the program began, primarily in response to scholars’ feedback. Directed readings, didactic lectures, group discussions, in-person workshops, and experiential learning through completing an individual research project using JHS data were the primary approaches to meeting these objectives.

While our novel program successfully met its objectives of training doctoral level students from underrepresented populations in cardiovascular epidemiology, UMMC GTEC nonetheless encountered significant barriers and challenges, requiring continuous review and refinement. Delivering a focused training program to multiple cohorts of doctoral students from various academic programs, both from research and clinical disciplines, across Mississippi was unexpectedly challenging. More than 75 % of the enrolled scholars were in professional or clinical doctoral programs, rather than research-oriented doctoral programs (e.g., medical, clinical psychology, basic laboratory science, and clinical pharmacy programs), therefore having little academic exposure to epidemiological and research methods. This affected scholars’ knowledge and use of epidemiologic research terminology; awareness of different approaches to human subjects study design, hypothesis development, and statistical analysis; exposure to and use of existing individual-level data; and scientific writing. As a result, the “complementary” nature of the program also had to adapt to these divergent levels of skills and knowledge: for scholars from stronger epidemiological and research methods backgrounds, the program aligned well with their preparation and primary doctoral studies and enriched their doctoral program; for others who were underprepared for epidemiologic research, much of UMMC GTEC’s content was completely new information, requiring a heavier “lift” for them.

The scholar mix and predominance of clinical and professional doctoral students may have contributed to some of the significant quantitative declines in career interest and outcome expectations at the completion of the program. The scholars’ variability required a more individualized and intensive approach to the mentoring process, with increasingly high levels of mentor assistance needed to produce scholarly deliverables and decreasing overall success with the peer-review process. Taken together, while their self-efficacy significantly improved and they saw the benefit of the program, some scholars also did not see the continuation of this type of work as desirable for their careers and future interest. Given the small analytic sample, a sub-group analysis of career interest and outcome expectations among those from professional or clinical doctoral programs compared to those from research-oriented doctoral programs was not feasible.

Given the multidisciplinary backgrounds of the scholars, an overview of epidemiology and public health needed to be provided for each cohort. The mentoring team incorporated the epistemological underpinnings of epidemiology set forth by Keyes and Galea (Keyes & Galea, 2014). Training in public health knowledge is helpful for students of all backgrounds, especially among those in behavioral and medical sciences, as they often do not have this general training (Hovell et al., 2008). In the qualitative program evaluation, training in epidemiology was reported as a highly valued aspect of GTEC. Many scholars reported increased knowledge of general epidemiological terms and how to apply these concepts to cardiovascular health. Particularly considering the level of preparation of our trainees when entering the program, we provided a significant enhancement to their understanding of, and capacity for research and pursuit of further training and careers in biomedical research. Supporting evidence for this conclusion is evident in the number of GTEC graduates who have continued research in medical residency or post-doctoral programs.

The UMMC GTEC mentors worked effectively with each scholar at their respective levels of ability and interest. Although UMMC GTEC did not empirically measure the knowledge that scholars gained, either through formative or summative examination or assessment, the scholars’ perception of their learning through the program was generally positive and indicative of good feasibility and success in meeting this objective.

The UMMC GTEC’s original intent was to meet the third objective of producing research deliverables and participating as members of the scientific community by each scholar co-authoring *two* manuscripts by the time they graduated from UMMC GTEC. In addition to this publication goal, the UMMC GTEC mentors were expected to produce two collaborative publications annually. Meeting this level of productivity proved to not be feasible, particularly considering the policies undergirding the use of JHS data, which require multiple rounds of review and approval from PPS, as well as the unique difficulties for collaboration that COVID presented for almost two years.

After the first cohort, the expectation for publications was revised to one manuscript per scholar during their 2-year program. However, even with this revision, the total average time to complete the multiple steps necessary to finalize an original manuscript proposal, gain project approval from PPS, receive the data, prepare and analyze the data, write the manuscript, receive PPS approval to submit the manuscript to a peer-reviewed journal, and subsequently submit the manuscript exceeded two years. Thus, the feasibility of manuscript publication with JHS data during the training program was quite low. This meant that to produce a scholarly publication, the scholars and their mentors had to continue working together beyond formal program participation. For most scholars, given their responsibilities to their primary doctoral programs for completion of a dissertation for graduation and/or moving into postdoctoral, academic, or clinical positions after completing their degree, continuation of this work was a particular challenge. While many scholars’ projects have resulted in conference abstracts, most did not move forward to external dissemination in a peer-reviewed journal.

The challenge of producing a peer-reviewed manuscript during the training period is not unique to just this program. Other research training programs have reported such barriers, from data acquisition to analysis to time management, to completing projects (Johnson, 2022). The successes in abstract submission and conference presentation for 15 of the 22 UMMC GTEC graduates demonstrate that the feasibility of submitting abstracts during the scholars’ two-year program is high, but the actual presentation at a conference also typically occurs after the program has ended (Byars-Winston et al., 2011).

The fourth objective, to encourage students to pursue future training and biomedical careers, is encompassed in the approach to meet the prior three objectives. Additionally, the career coaching and mentor discussions about future avenues after completion of their doctoral degree allowed the scholars to consider different perspectives and to be exposed to alternative career paths than what they may learn about from their primary degree faculty. Finally, the near-peer interactions between the GTEC graduates and later cohorts also provide valuable information to shape the scholar’s vision of their future. This component has been a success, with more than 50 % of all GTEC graduates who have also graduated from their doctoral programs continuing with additional training.

Note that the results presented here are from a formal program evaluation as a quality assurance aspect of the program, which was not designed as research. The interpretation of the findings is limited by the small sample size; we were unable to stratify based on the scholar’s primary program type. Therefore, it is difficult to fully understand whether the students from research-based doctoral programs differed in their experience and outcomes from the clinical doctoral students.

## 6. Conclusion

The UMMC GTEC Program enrolled 38 students since 2019, 22 of whom completed the program, and 12 of whom remained active at the time of this writing. This complementary research mentoring program provided a select component of Mississippi doctoral students with enhanced research training and opportunities to contribute to and join a community of research scholars in cardiovascular epidemiology. The program found great overall success in meeting its objectives, and the scholars benefited from participation in many expected and surprising ways. Overall, the results from the qualitative and quantitative evaluation of UMMC GTEC scholars, the number of scientific deliverables produced by the scholars and their mentoring teams, and the number of graduates continuing their biomedical training after completion of their doctoral programs provide evidence that the UMMC GTEC positively contributed to training the next generation of cardiovascular epidemiology researchers in Mississippi.

## Supplementary Material

1

2

3

## Figures and Tables

**Table 1:** Qualitative themes and supporting evidence.

**Theme 1:** Empathetic and accessible mentorship fosters positive research experiences among doctoral and graduate students.
*Our mentors always made a safe space for us. They were always available. I could go to them with career-based questions, and they were always down to talk about things like that. It was very helpful to know that I had people I could bounce questions off like that as well*. Participant 008
*I would say the most important aspect of the mentor mentee relationship was communication and transparency, being honest about a lot of things. [My mentor] kept up with each and every one of our products and helped with sending articles in the middle of a random week saying, ‘Hey, just found this [article] check this out.’ I think, for me personally, the [GTEC] mentor-mentee experience is what I would brag about to anybody outside of my PhD program*. Participant 013
*Seeing the disagreement between mentors on how you should write, where you should put something, and learning when disagreement is okay – different researchers do have different ways of doing things, and there are some things where there is no right or wrong way of doing things – seeing these different points of view helped me increase my confidence in feeling like I could navigate these things*. Participant 004
**Theme 2:** Mentored experiential research opportunities increase self-efficacy in research and scientific communications.
*[GTEC] helped me understand the scope of research, how to conduct research, especially within the Jackson Heart Study, and how to disseminate that information and approach it from a statistical perspective. I think from a research perspective; I think the stats knowledge was invaluable. I think that was a, a major part of knowledge that I had gained, and how to translate [ideas] to scientific writing as we built the proposal*. Participant 004
*[I received] a lot of the feedback from mentors on the way that I write scientifically. I think that now that I’ve been through the program, my scientific writing skill set has improved a good bit. I’ve learned how to be more concise and clearer and to use words that have a little bit better precision when I write*. Participant 009
*GTEC has gotten me to think outside the box as a researcher. The nomenclature like the use of ‘exposures’ and ‘outcome variables.’ Understanding the use of covariables in your research has helped me to understand other things. I also had the greatest amount of growth as a writer*. Participant 013
*I’ve learned how to be a lot more flexible and communicate with people a little bit better about research. I am also gaining an appreciation of other people’s perspectives. I am learning how to take criticism and not take it as a negative thing, but to move forward with it and improve the work. That’s been a really good skill, too. I think I’ve learned how to be a better team member through GTEC*. Participant 011
**Theme 3:** Research is a lengthy and rigorous process.
*I’ve learned how to get my thoughts on paper and go back to clean it up. I need the best written, thought-out information. I’m learning the art of writing and a patience that comes in writing*. Participant 014
*It took me so long to even get my proposal written and submitted. Once it was submitted, I got two comments. One was that [PPS] wanted to change my title because it didn’t fit my hypothesis. The other was that [PPS] wanted me to add in some kind of statistical analysis, which I didn’t know anything about. That felt really good because it took me so long to submit – so much reading, so much writing to even get it submitted and when it finally did, it was just a couple comments. My mentors were very supportive of that. They said this was probably the best result [from PPS] that we could have gotten. It felt like it was all worth it, the struggle for two years was worth it. That was probably the best part*. Participant 005
*I just got my proposal approved, so I haven’t done a whole lot of the data analysis or actual paper writing. That was something that I really did want from this experience but haven’t gotten yet. And that’s mainly because I think I had four different proposals essentially. I continuously asked questions that couldn’t be answered with the data set and so that was a little frustrating*. Participant 012
*Research, I did not know that it took that much to receive secondary data. We don’t have to do all of that [in basic science]. It’s kind of like, we just write the protocol, it gets approved, then we do the experiments. I learned that I have to go through manuscript proposals, make sure my idea doesn’t overlap with previous manuscripts. I learned how to write a manuscript proposal effectively, and then I learned to wait to hear back [from PPS] and then I also learned to write the abstract in a different type of way. Abstracts are different than a manuscript*. Participant 016
**Theme 4:** Participation in a mentored research training program supports scholars’ aspirations for a career in research.
*It did help me realize that this is something that I want to continue doing, even if it is difficult sometimes. So, I’d say that I’m leaning more towards a 4 [out of 5], I agree that the program did help me realize my career aspirations.* Participant 005
*I’m considering applying to postdoctoral programs that focus on health policy and getting more experience in writing grants. Maybe moving on to a nonprofit organization around health care quality, or access to health care, or maybe looking at an academic position*. Participant 009
*I’m actually considering doing a discipline shift. I want to do more of sort of what we’ve done in GTEC. I’m looking at a few T32 training grant programs that extend this into cardiovascular epidemiology. I’m really hoping that I might be able to get into those to really develop my own research projects that I can continue as an academic researcher*. Participant 017
**Theme 5:** Science identity emerges through socialization into the norms and discourse practices of science.
*I feel like there’s cohesion in GTEC. Even if me and an individual weren’t working on the same topic – mine was genetics and there’s might be activity – I felt like there was still enough overlap that we could still have a conversation and discuss things. I felt like there was more of a community there*. Participant 003
*There’s something to be said about the network that you make among your peers. You make connections and collaborations with researchers and those that are your mentors. But there’s something to be said about your cohort team. People who are in a very similar state that you are as far as academia doing a fellowship program – that could be one of the best experiences of GTEC, really getting to learn folks*. Participant 012
*I think the best part was being able to meet other students from different universities and to work along with them, where we were both working on research but being able to learn from them. Also getting to know them and becoming friends with them as well. That’s been like networking, and then from there, you actually have these friends that you can kind of rely on later on, too.* Participant 010

**Table 2:** A side-by-side joint display of UMMC GTEC evaluation findings.

	Baseline M (SD)	Post M (SD)	MD (SD)	t-stat, df	*P*	Qualitative themes	Integration
**Mentor influence**							
Vicarious learning	2.93 (0.96)	3.00 (1.15)	0.08 (1.39)	0.85, 19	0.406	Empathetic and accessible mentorship fosters positive research experiences among doctoral and graduate students.	Converge
Social persuasion	3.20 (0.97)	2.80 (1.21)	−0.40 (1.18)	1.89, 19	0.074		Diverge
Affective response*	2.08 (1.03)	1.95 (1.05)	−0.13 (1.11)	1.04, 19	0.310		Converge
**Self-efficacy**							
Scientific writing	2.51 (0.71)	2.97 (0.51)	0.46 (0.55)	3.98, 19	0.001	Mentored experiential research opportunities increase self-efficacy in research and scientific communications.	Converge
Oral presentations	2.57 (0.79)	3.19 (0.69)	0.62 (0.76)	3.93, 19	0.001		Converge
Scientific conversations	2.29 (0.72)	2.94 (0.45)	0.64 (0.63)	4.87, 19	< 0.001		Converge
**Career outcome expectations**							
Positive	3.30 (0.46)	3.00 (0.48)	−0.30 (0.44)	3.35, 19	0.003	Research is a lengthy and rigorous process.	Converge
Negative*	1.36 (0.88)	1.57 (0.87)	0.21 (0.73)	1.68, 19	0.110		Converge
**Career interests**							
Scientific writing	3.74 (0.38)	3.11 (0.86)	−0.63 (0.85)	3.61, 19	0.002	Participation in a mentored research training program supports scholars’ aspirations for a career in research.	Diverge
Oral presentations	3.34 (0.55)	2.86 (0.91)	−0.48 (0.72)	3.26, 19	0.004		Diverge
Scientific conversations	3.33 (0.49)	3.01 (0.70)	−0.31 (0.79)	2.13, 19	0.046		Diverge
**Science identity**	2.58 (1.18)	2.92 (0.80)	0.33 (1.38)	1.51, 19	0.147	Science identity emerges through socialization into the norms and discourse practices of science.	Converge

* Affective response mentor influence and negative career outcome expectations were asked in a negative direction, making decreased scores indicative of a positive outcome. All other questions were asked in a positive direction, with increased scores indicating a positive outcome.

M = mean; SD = standard deviation; MD = mean difference; t-stat = t-statistic for paired *t*-test; df = degrees of freedom

## Appendix A. Supporting information

Supplementary data associated with this article can be found in the online version at doi:10.1016/j.evalprogplan.2025.102606.
