# Supplementary material for: Outcomes and evaluation of a National Institutes of Health funded training program for doctoral students: The Jackson Heart Study Graduate Education and Training Center at the University of Mississippi Medical Center
Source: Eval Program Plann. Author manuscript; Available in PMC 2026 Jun 17. (PMC13274377; doi:10.1016/j.evalprogplan.2025.102606)
Supplement: 1 [file NIHMS2175185-supplement-1.docx]

| **S1 Table: Survey Instruments** | | | | | |
| --- | --- | --- | --- | --- | --- |
| Scientific Writing Self-efficacy * | | | | | |
| Rate your level of confidence (even if you have never done it yet) in your ability to... | | | | | |
|  | Very Insecure | Insecure | Neither Confident nor Insecure | Confident | Very Confident |
| 1. Excel in scientific writing tasks (e.g., abstracts, manuscripts). |  |  |  |  |  |
| 2. Deal with a lack of mentor support in scientific writing. |  |  |  |  |  |
| 3. Complete a writing task in the time allowed. |  |  |  |  |  |
| 4. Write and submit an abstract to a scientific meeting. |  |  |  |  |  |
| 5. Write a first draft by yourself of a manuscript intended for publication |  |  |  |  |  |
| 6. Write using correct grammar. |  |  |  |  |  |
| 7. Manage any anxiety you may have about your writing ability. |  |  |  |  |  |
| 8. Use the expected scientific style when writing. |  |  |  |  |  |
| 9. Continue to revise a manuscript multiple times after receiving negative feedback from your mentor or reviewers. |  |  |  |  |  |
| 10. Need minimal help because my writing skills are strong enough. |  |  |  |  |  |
| Scientific Oral Presentation Self-efficacy | | | | | |
| 11. Excel in giving scientific presentations (i.e., you usually receive high praise for your presentations from your mentor or the audience). |  |  |  |  |  |
| 12. Give a scientific talk to a lay audience (e.g., high school students, cancer patients). |  |  |  |  |  |
| 13. Give an oral presentation at a scientific meeting. |  |  |  |  |  |
| 14. Require little to no assistance with my speaking and presenting skills. |  |  |  |  |  |
| Scientific Conversation Self-efficacy | | | | | |
| 15. Defend your point of view convincingly in a scientific discussion, in spite of a negative response from others. |  |  |  |  |  |
| 16. Effectively answer questions from the audience at a scientific Meeting. |  |  |  |  |  |
| 17. Speak using correct grammar without rehearsing. |  |  |  |  |  |
| 18. Manage worries you may have about your pronunciation, accent, vocabulary, grammar, or style of speaking. |  |  |  |  |  |
| 19. Ask a question or add a comment during a meeting or discussion in your own lab or research group. |  |  |  |  |  |
| 20. Ask a question in front of the audience after a presentation at a national scientific meeting. |  |  |  |  |  |
| 21. Use the expected scientific style when speaking. |  |  |  |  |  |
| 22. Introduce yourself and your research concisely and effectively to other professionals. |  |  |  |  |  |

| Respond to the following statements with the response that best fits you or your situation. † | |
| --- | --- |
| Vicarious Learning | |
| 23. My primary research mentor showed me how to conduct a research procedure. | Strongly Disagree |
|  | Disagree |
|  | Neither Agree or Disagree |
|  | Agree |
|  | Strongly Agree |
| 24. I look up to my research mentor as a career role model. | Strongly Disagree |
|  | Disagree |
|  | Neither Agree or Disagree |
|  | Agree |
|  | Strongly Agree |
| Social Persuasion | |
| 25. My research mentor encouraged me to pursue a research science career. | Strongly Disagree |
|  | Disagree |
|  | Neither Agree or Disagree |
|  | Agree |
|  | Strongly Agree |
| 26. My research mentor told me I have the ability to be a scientist. | Strongly Disagree |
|  | Disagree |
|  | Neither Agree or Disagree |
|  | Agree |
|  | Strongly Agree |
| Affective/Emotional Arousal | |
| 27. I felt nervous when conducting research. | Strongly Disagree |
|  | Disagree |
|  | Neither Agree or Disagree |
|  | Agree |
|  | Strongly Agree |
| 28. I felt anxious about my ability to do research. | Strongly Disagree |
|  | Disagree |
|  | Neither Agree or Disagree |
|  | Agree |
|  | Strongly Agree |

| Career Outcomes Expectations * | | | | | |
| --- | --- | --- | --- | --- | --- |
| My work to achieve high performance in scientific writing and speaking will... | | | | | |
|  | Strongly Disagree | Disagree | Neither Agree nor Disagree | Agree | Strongly Agree |
| 29. Allow me to obtain a highly desirable academic faculty position. |  |  |  |  |  |
| 30. Be necessary for me to be recognized as an expert in my research area. |  |  |  |  |  |
| 31. Be critically important for me to become a successful independent investigator. |  |  |  |  |  |
| 32. Make me feel good about myself. |  |  |  |  |  |
| 33. Inspire me to do great work. |  |  |  |  |  |
| 34. Make me feel confident and secure about my future career. |  |  |  |  |  |
| 35. Deprive me of time with family and friends. |  |  |  |  |  |
| 36. Lead to chronic stress, anxiety, and worry in my life. |  |  |  |  |  |
| 37. Cause me to lose sleep. |  |  |  |  |  |
| 38. Make me become angry and frustrated. |  |  |  |  |  |
| 39. Cause my physical health to become poor. |  |  |  |  |  |

| Interest in Scientific Writing * | | | | | | | | | | | | | | | |
| --- | --- | --- | --- | --- | --- | --- | --- | --- | --- | --- | --- | --- | --- | --- | --- |
| During my current training period, I am interested in... | | | | | | | | | | | | | | | |
|  | | Strongly Disagree | | | Disagree | | | Neither Agree nor Disagree | | | Agree | | | Strongly Agree | |
| 40. Writing first-author manuscripts for submission to journals. | |  | | |  | | |  | | |  | | |  | |
| 41. Writing manuscripts with other authors. | |  | | |  | | |  | | |  | | |  | |
| 42. Writing and submitting abstracts to scientific meetings. | |  | | |  | | |  | | |  | | |  | |
| 43. Creating a poster of my work. | |  | | |  | | |  | | |  | | |  | |
| Interest in Scientific Oral Presentation | | | | | | | | | | | | | | | |
| During my current training period, I am interested in... | | | | | | | | | | | | | | | |
|  | Strongly Disagree | | | Disagree | | | Neither Agree nor Disagree | | | Agree | | | Strongly Agree | | |
| 44. Giving an impressive oral presentation at a national scientific meeting. |  | | |  | | |  | | |  | | |  | | |
| 45. Presenting my poster formally to a seated audience at formally to a seated audience at a scientific meeting. |  | | |  | | |  | | |  | | |  | | |
| 46. Presenting a summary and leading the discussion of an article for fellows' "journal club". |  | | |  | | |  | | |  | | |  | | |
| 47. Explaining my poster informally during a poster session during a scientific meeting. |  | | |  | | |  | | |  | | |  | | |
| Interest in Scientific Conversation | | | | | | | | | | | | | | | |
| During my current training period, I am interested in... | | | | | | | | | | | | | | | |
|  | | | Strongly Disagree | | | Disagree | | | Neither Agree nor Disagree | | | Agree | | | Strongly Agree |
| 48. Asking questions of a presenter at a scientific meeting. | | |  | | |  | | |  | | |  | | |  |
| 49. Actively participating in group scientific discussions. | | |  | | |  | | |  | | |  | | |  |
| 50. Making an outstanding impression introducing myself and my research to various individuals. | | |  | | |  | | |  | | |  | | |  |
| 51. Being able to express my ideas eloquently to a variety of audiences. | | |  | | |  | | |  | | |  | | |  |

| Research Science Career Outcomes Expectations † | | | | | |
| --- | --- | --- | --- | --- | --- |
| A research science career would allow me to... | | | | | |
|  | Strongly Disagree | Disagree | Neither Agree nor Disagree | Agree | Strongly Agree |
| 52. Do work that makes a difference in people's lives or society |  |  |  |  |  |
| 53. Do work that I find satisfying |  |  |  |  |  |
| 54. Go into a field with high employment demand |  |  |  |  |  |
| 55. Get respect from other people |  |  |  |  |  |
| 56. Earn an attractive salary |  |  |  |  |  |

| Science Identity † | | | | | |
| --- | --- | --- | --- | --- | --- |
| During my most recent research experience, I... | | | | | |
|  | Strongly Disagree | Disagree | Neither Agree nor Disagree | Agree | Strongly Agree |
| 57. Felt like a scientist |  |  |  |  |  |
| 58. Interacted with scientists from outside my school |  |  |  |  |  |
| 59. Felt part of a scientific community |  |  |  |  |  |

* Anderson CB, Lee HY, Byars-Winston A, Baldwin CD, Cameron C, Chang S. Assessment of Scientific Communication Self-Efficacy, Interest, and Outcome Expectations for Career Development in Academic Medicine. J Career Assess. 2016 Feb 1;24(1):182-196. doi: 10.1177/1069072714565780. Epub 2015 Jan 6. PMID: 26924920; PMCID: PMC4764330.

† Adapted from: C. B. Anderson, H. Y. Lee, A. Byars-Winston, C. D. Baldwin, C. Cameron, and S. Chang, "Assessment of Scientific Communication Self-efficacy, Interest, and Outcome Expectations for Career Development in Academic Medicine," Journal of Career Assessment, vol. 24, no. 1, pp. 182-196, 2016, doi: 10.1177/1069072714565780.
