# Supplementary material for: Outcomes and evaluation of a National Institutes of Health funded training program for doctoral students: The Jackson Heart Study Graduate Education and Training Center at the University of Mississippi Medical Center
Source: Eval Program Plann. Author manuscript; Available in PMC 2026 Jun 17. (PMC13274377; doi:10.1016/j.evalprogplan.2025.102606)
Supplement: 2 [file NIHMS2175185-supplement-2.docx]

| **Supplemental Table 2. Qualitative Themes and Supporting Data** | | |
| --- | --- | --- |
| **Theme 1:** Empathetic and accessible mentorship fosters positive research experiences among doctoral and graduate students. | | |
|  | *Vicarious learning* | |
|  |  | During [mentor] meetings, we would discuss pretty much anything that I needed to discuss at that time. It was really me directing the meeting, which I liked. I was able to show them what I had at the time, and they could interpret whether or not what I was doing was correct or incorrect. They would provide feedback at the meeting so; I was able to write everything down. In certain scenarios where we weren't able to meet, I was able to email questions and if those questions couldn't be answered over email, then we would set up an impromptu meeting a couple of days later so I could navigate next steps.  Seeing the disagreement between mentors on how you should write, where you should put something, and learning when disagreement is okay – different researchers do have different ways of doing things and there are some things where there is no right or wrong way of doing things – seeing these different points of view helped me increase my confidence in feeling like I could navigate these things.  I did receive a lot of feedback on the work that I did. We would exchange comments on written work through email and go back and forth to identify a happy middle ground on everyone's comments and find ways to address everyone's issues. That works really well for me. I do really well in a self-driven environment. I think emailing and having deadlines that either I set or were set by conferences, that was really helpful for me.  The feedback via email works just fine for presentations. A lot of times we would have meetings, and I would practice the presentations, and [mentors] would give me feedback afterwards about what I should do differently or things to change or consider. I felt that that was really helpful, too. I felt like it met my needs moving through the program.  [I learned] how to receive feedback and how to [respond to] feedback in a timely manner and you can move forward with things. That was a big takeaway in general from GTEC.  I can put the work in. There's sometimes I will get to a place of having knowledge of what I need, but there's like a lifelong student mindset that [mentors] have. I don't like sitting there, learning, and teaching myself. I'd rather be taught things, but [mentor] is always reading, [they’ve] never stopped reading. |
|  | *Social persuasion* | |
|  |  | I feel like [mentor-A] really pushed me to achieve more than I thought I could. Once I got the data for my project, [mentor-A] really pushed me and was really big on setting up the meetings with me. [Mentor-B] and I started meeting regularly to get the project moving and identifying different conferences and then encouraging me to apply for these different conferences. I presented my research at 2 different conferences.  Last week in our meeting, I was talking about a job I was considering applying for. I shared that I didn't know if it was an assistant professor position that I really wanted, because it was a teaching position, there was no research involved. We talked about that, and [they] shared with me ideas like, if I pursued this position and got this position, how I could make my way into research in that department after being hired. If I wasn't in GTEC, I wouldn't get that perspective and valuable advice.  A lot of the time the mentor, I would send them whatever I wrote, and the mentors would provide their comments. Their feedback, which I found was very helpful, in some instances, they would even just flat out say this is a better way to write this, which is very helpful.  I guess the writing part I like. I made like the 2nd most progress in our cohort [on writing]. So, it was helpful to have [mentors] meet with me every week for 30 minutes.  I would say the most important aspect of the mentor mentee relationship was communication and transparency, being honest about a lot of things. [Mentor] kept up with each and every one of our products and helped with sending articles in the middle of a week saying, ‘Hey, just found this [article] check this out.’ For me personally the mentor-mentee experience is what I would brag about to anybody outside of my PhD program. |
|  | *Affective response* | |
|  |  | Our mentors always made a safe space for us. They were always available. I could go to them with career-based questions, and they were always down to talk about things like that. It was very helpful to know that I had people I could bounce questions off like that as well.  I think the mentors were very on top of things. [Mentor] sent calendar invites and we would meet. Everything was well structured in my opinion when it came to what I needed to do for my manuscript. There were times that I was frustrated and confused, but I think that I can be attributes to a lack of knowledge base. Once I needed help, it was there. That was always really, really good.  Overall, the feedback from everyone was good. I say that some of it was positive, but they all had different ways of doing it. It was always good to get feedback from [mentor], the ways that they could really take their background in ethics, and just overall as a researcher, [they are] really, really amazing. On top of the fact that [they] would go out of [their] way to find the time. I still don't know how [they] do all of that.  I personally appreciate a direct approach to feedback which [mentors] have given me. I will say [mentor] was an excellent addition to the team, an absolute excellent addition. The meetings that I had with [mentor] to think through the stats, we would both sit and think through the stats together and in between meetings, [mentor] would go off and think about something and then come back and ask what I thought about it. [Mentor] was incredibly patient with helping to guide me. |
| **Theme 2:** Mentored experiential research opportunities increase self-efficacy in research and scientific communications. | | |
|  | *Research conduct* | |
|  |  | I feel more confident going forward that I can do statistics because now I have done it once at least. And then again, with writing, [mentors] helped me get two manuscripts under my belt that I would not have gotten if I wasn't in GTEC. I got feedback from so many people, and I wouldn't have gotten their perspective if I wasn't in the program. So, statistics and writing I feel much more confident about because of GTEC.  I can't think of anything that I didn't get [from GTEC]. I went into it not knowing what to expect because I knew there was a lot that I didn't know. So going into it, I didn't have certain expectations. But I feel like coming out of GTEC, I have been taught more than what I thought that I would know.  In my own PhD work, there's a big emphasis on the technical stuff, doing experiments and carrying them out. But I felt with GTEC there was much more emphasis on the design element of [research]. That's been very helpful for me. It's allowed me to really think a lot more about the type of research I'm doing, and what kind of questions I'm asking, that I don't really get from my PhD program. |
|  | *Epidemiology and biostatistics* | |
|  |  | [GTEC] did help me to get some background on how to approach human data. I will say that it did help me in that aspect of just learning how to translate epidemiology data.  I learned how to code with STATA and that has been amazing. Not an easy feat. I've gained a real appreciation for and understanding of the limitations of the data you work with and how the data shouldn't necessarily shape your research question and its outset, but it definitely drives your question. And I think that I've gained a much better appreciation of that through this program.  [GTEC] helped me understand the scope of research, how to conduct research, especially within the Jackson Heart Study, and how to disseminate that information and approach it from a statistical perspective. I think from a research perspective; I think the stats knowledge was invaluable. I think that was a major part of knowledge that I had gained, and how to translate [ideas] to scientific writing as we built the proposal.  We had [mentor] in the winter session go over different R statistic software and that was new to us when we originally started with STATA in my 1st year. So, even just getting that experience with different stats software, it's just adding those tools to our tool belt.  I think specific training had to do with stats. My cohort started off with STATA – and another thing that I was trying to improve was any kind of coding experience for my clinical teaching endeavors and I know in STATA, you can kind of have a coding aspect to it. So, that was the main thing that I wanted to get out of [GTEC] and then whenever we pivoted towards using R, that was even better.  I had a very weak stats background, so I was hoping that I would get that, and I got it more than I hoped for. I'm coming out of [GTEC] forever grateful to [mentor] for sitting hours upon hours upon hours on Zoom with me going over stats.  The biggest point was learning statistical methods from an epidemiological standpoint, which is very different than what I do, but it's just as important. So that was super helpful.  Primarily working with large datasets is not really something that I do in my PHD. [GTEC] works with something that I really wanted exposure with, especially working in R. I've gained a lot from this, that's been a really good experience. |
|  | *Scientific writing* | |
|  |  | I think the biggest enhancement that GTEC had was in the writing process, getting feedback from different people. I remember one of my mentors was nit-picky, but not in a negative way. In my title, I said, ‘in African Americans’ instead of ‘among African Americans’. It was little stuff like that, I would have never realized that it wasn't the appropriate term. Getting that feedback and having them kind of nitpick at different words was very helpful for me.  I really liked the multiple rounds of feedback that I got from the entire team of mentors cause everyone finds different things in your writing. I was happy to have criticism of my writing because I wasn't getting that in my home department. So [GTEC] did increase my confidence in that way.  [I received] a lot of the feedback from mentors on the way that I write scientifically. I think that now that I've been through the program, my scientific writing skill set has improved a good bit. I've learned how to be more concise and clearer and to use words that have a little bit better precision when I write.  In terms of writing a manuscript, I'm still working on that now, but getting to the point after having a question to getting the data and doing the analysis, being able to put that into a manuscript – at our last meeting, [mentors] went into a lot of depth about how to write a manuscript; that was very helpful because I haven't done it before. Knowing the steps and knowing what [a manuscript] should look like was a big help to get me where I am now.  Especially when dealing with human data there are certain types of language that is acceptable and is not acceptable. Being as objective as possible and interpreting the data and being mindful of who my audience is while writing these papers is important.  The program also really helped with writing certain grants. A lot of the NIH funding is going towards translational research. It's a lot easier to translate the research when you have some of that background in epidemiology.  That’s how GTEC helped me with research, as a writer. Besides that, you learn writing perspectives different than ones you have right now and for me, I think no doubt it is one of the biggest things I have taken away to use in the future.  With the manuscript proposal, it was just it's kind of different writing from basic science writing. I did learn that while I already knew that specific words all have different weights, trying to be more mindful of the words you use developed for me through GTEC.  GTEC has gotten me to think outside the box as a researcher. The nomenclature, the use of ‘exposures’ and ‘outcome variables.’ Understanding use of covariables in your research has helped me to understand other things. I also had the greatest amount of growth as a writer.  Scientific writing is a different kind of writing than I do but just being able to hear the feedback from the mentors, especially those who are at UMMC and are in the world of epidemiology was really helpful. I would phrase something, and [mentor] would be like, this actually means something completely different than what you're talking about. And I was like, ‘Ok, cool’. But GTEC has definitely sharpened my scientific skills. |
|  | *Oral presentations* | |
|  |  | I did get some verbal feedback from two other mentors. Essentially my slides were not very well organized, that was some of the feedback that I got. It kind of helped me from that point forward to kind of think about sequence in my slides in a more appropriate manner that made sense. So, that was one of my biggest takeaways.  I presented the research at 3 different platforms and getting that comfortability of talking to people, speaking, and presenting – I think that a really helpful part of GTEC that's contributed to my growth tremendously as a graduate student.  Personally, I've worked with [mentor], that's been an incredible relationship and preparing for presentations and some of [their] expertise in that regard. Also just working with all these other professors or these other schools has been great. I have no letters next to my name, right? That imposter syndrome that seeps in but, I’m networking with all these great individuals and being treated as an equal. |
|  | *Scientific conversations* | |
|  |  | I've learned how to be a lot more flexible and communicate with people a little bit better about research. I am also gaining an appreciation of other people's perspectives. I am learning how to take criticism and not take it as a negative thing, but to move forward with it and improve the work. That's been a really good skill, too. I think I've learned how to be a better team member through GTEC.  I have this specific research question in the field that I'm in and having really good conversation regarding those questions, or we go to a conference and talk about different posters or presentations and being able to have that back and forth has been really great.  I will say the biggest [takeaway from GTEC] is the writing and just being affiliated with the Jackson Heart Study. This past week, I had an interview, and I was able to talk about the [Jackson Heart Study]. [The interviewers] have a longitudinal study that focuses on Alzheimer’s disease, so I was able to talk about it because I've read so much about the Jackson Heart Study so just having that general knowledge has been beneficial. |
| **Theme 3:** Research is a lengthy and rigorous process. | | |
|  | *Positive career outcome expectations* | |
|  |  | I've learned how to get my thoughts on paper and go back to clean it up. I need the best written, thought-out information. I'm learning the art of writing and a patience that comes in writing.  It took me so long to even get my proposal written and submitted. Once it was submitted, I got two comments. One was that [PPS] wanted to change my title because it didn't fit my hypothesis. The other was that [PPS] wanted me to add in some kind of statistical analysis, which I didn't know anything about. That felt really good because it took me so long to submit – so much reading, so much writing to even get it submitted and when it finally did, it was just a couple comments. My mentors were very supportive of that. They said this was probably the best result [from PPS] that we could have gotten. It felt like it was all worth it, the struggle for two years was worth it. That was probably the best part.  Research, I did not know that it took that much to receive secondary data. We don't have to do all of that [in basic science]. It's kind of like, we just write the protocol, it gets approved, then we do the experiments. I learned that I have to go through manuscript proposals, make sure my idea doesn’t overlap with previous manuscripts. I learned how to write a manuscript proposal effectively, and then I learned to wait to hear back [from PPS] and then I also learned to write the abstract in a different type of way. Abstracts are different than a manuscript.  With GTEC, because we have to design our own projects, we had to really dive into the literature and in some respects, become our own sort of mini experts in this topic. It really, really taught me the importance of really diving into the literature and understanding what you're trying to look at and coming up with good questions.  As it relates to knowing how to interpret epidemiological data and how to connect that with basic [science] research. I think that the fact that the question we had to choose, had to relate in some way to our research. [It] really helped seeing the connection with what my dissertation question is in an animal model how that can be tied back in some form to the epidemiology data that we're looking at.  We're still like writing up the manuscript, but I realized I'm going to add an extra year to my PhD program. I was going to do 3 years. I'm going to go into 4 years now. I think [GTEC] just helped me realize there's still a lot learning the ropes. But moving forward, I'm gonna stay in the academia world at least getting a PhD for a couple more years. I'm interested in exercise and physiological adaptations to exercise particularly in extreme environments. I'm hoping to go into working either as a post doc doing research or working for a company that can assist athletes who do extreme sports. |
|  |  |  |
|  | *Negative career outcome expectations* | |
|  |  | I remember [mentor] hadn't looked at any of my work, but in that moment, they realized that the question I was trying to answer couldn't be answered. I had to go back to the drawing board from there. But my primary mentors had read my paper, and we had been going back and forth talking about this for months, but when I presented, they realized, ‘What you're asking can't be answered so, you might need to start over’. That was a big hurdle because at that point I had to start all over. Then when I met with my primary mentors they were like, ‘this isn't a start over, you can still use some of the information that you already have you just can't ask that question.’  I just got my proposal approved, so I haven't done a whole lot of the data analysis or actual paper writing. That was something that I really did want from this experience but haven't gotten yet. And that's mainly because I think I had four different proposals essentially. I continuously asked questions that couldn't be answered with the data set and so that was a little frustrating.  I would have liked to have gotten farther in my process, at least in the paper writing stage, because you need a lot of feedback there. At least the first draft or something, but that's on me as well.  I never presented anything. I definitely wanted to be able to present and there were things that my mentors could not predict, which was that PPS was gonna say you can't use this data, even after they already said we could use it. So, I was highly frustrated by that. Now, I'm working on somebody else's paper and currently gonna be 3rd or 4th author on that paper, and I won't get a 1st author paper out of it.  You have to find a way to propose what you're interested in and then do a secondary analysis. That was very challenging and continues to be challenging to this point. [Research is challenging] when you're not used to being a part of the entire process. That was the biggest challenge. |
| **Theme 4:** Participation in a mentored research training program supports scholars’ aspirations for a career in research. | | |
|  | | It did help me realize that this is something that I want to continue doing, even if it is difficult sometimes. So, I'd say that I'm leaning more towards a 4 [out of 5], I agree that the program did help me realize my career aspirations.  I just had my interview [for a post doc] and, I'm completely switching fields so it's like clinical Alzheimer’s and stroke. So, I won't have to interact with animals or anything.  [In 5 years], I'll be starting the post doc, and around year 5, I will be starting my academic position, getting startup funds, and setting up my lab and team.  I'm considering applying to postdoctoral programs that focus on health policy and getting more experience in writing grants. Maybe moving on to a nonprofit organization around health care quality, or access to health care, or maybe looking at an academic position.  GTEC definitely supported my career aspirations. [GTEC mentors and faculty] had a bunch of career mentoring sessions. For me, personally, I love [mentor]. They was so great at merging both my personal and professional goals and seeing me, the full person who wants to have a full complete life and doesn't just look at me as a person to get me from X to Z. I'm doing a summer internship, and I put one of the mentors as a reference.  I think putting [GTEC] together with my experience during my doctoral program, my goal is to be in academia. Whether it's teaching intensive at an institution or research intensive, I feel like I'm very open to either, but the applied research side of things is what I really enjoy.  I'm actually considering doing a discipline shift. I want to do more of sort of what we've done in GTEC. I'm looking at a few T32 training grant programs that extend this into cardiovascular epidemiology. I'm really hoping that I might be able to get into those to really develop my own research projects that I can continue as an academic researcher.  I hope to be a pediatrician doing research.  I should complete my PhD by next year and I'm still researching to figure out which part of academia I want to be. What does it look like for me? Don’t know. But research for sure, just not sure what type of institution. |
| **Theme 5:** Science identity emerges through socialization into the norms and discourse practices of science. | | |
|  |  | Most immediately, my network, so my network has expanded a lot because of GTEC. I talked with previous graduates, [GTEC graduate] is a co-author on the abstracts I've been submitting and has been a big help. I also talked with [GTEC graduate], who has a postdoc, and we don't exactly share the same research field, but knowing that's something you can move forward to has been really helpful.  I think the best part was being able to meet other students from different universities and to work along with them, where we were both working on research but being able to learn from them. Also getting to know them and becoming friends with them as well. That's been like networking, and then from there, you actually have these friends that you can kind of rely on later on, too.  There's something to be said about the network that you make among your peers. You make connections and collaborations with researchers and those that are your mentors. But there's something to be said about your cohort team. People who are in a very similar state that you are as far as academia doing a fellowship program – that could be one of the best experiences of GTEC, really getting to learn folks.  I feel like there's cohesion in GTEC. Even if me and an individual weren't working on the same topic – mine was genetics and there's might be activity – I felt like there was still enough overlap that we could still have a conversation and discuss things. I felt like there was more of a community there.  I just feel more confident, overall, in my ability to do research. There was a good networking opportunity during the program, too. It was great having mentorship as well to talk about career type of stuff. I found that very beneficial.  GTEC provided me with a soft entry to research as an alternative way to start out and give me different perspectives because I'm going into exercise science. I don't do much with epidemiology. Working with a mentor, like a close team of mentors was appealing to me.  Learning how to form a research question was probably the hardest part, but being able to learn how to form a question and use the data that's already available to answer that question was how GTEC can help me reach my goal of being a physician scientist.  [Mentors] gave me a lot of opportunities to present at conferences by making me aware about what conferences there were. |
