# Supplementary material for: Outcomes and evaluation of a National Institutes of Health funded training program for doctoral students: The Jackson Heart Study Graduate Education and Training Center at the University of Mississippi Medical Center
Source: Eval Program Plann. Author manuscript; Available in PMC 2026 Jun 17. (PMC13274377; doi:10.1016/j.evalprogplan.2025.102606)
Supplement: 3 [file NIHMS2175185-supplement-3.docx]

| S3 Table. University of Mississippi Medical Center Graduate Training and Education Center Scholars and Areas of Research, The Jackon Heart Study (JHS) | | | | | | |
| --- | --- | --- | --- | --- | --- | --- |
| Scholar ID | **Start Date**  **Cohort #** | | **Scholar’s Institution** | **Major** | **Degree(s) Received (Year)** | **Area of JHS Research** |
| 1.1 | 2019  Cohort 1 | Mississippi State University | | Kinesiology & Exercise Science | BS (2015 Exercise & Movement  PhD (2020) Kinesiology | Sedentary Behavior & Metabolic Syndrome |
| 1.2 | 2019  Cohort 1 | UMMC | | Biostatistics & Data Science | BS (2015) Chemistry  MPH (2016) Epidemiology  PhD (2024) Population Health Science | Sleep and Mobility |
| 1.3 | 2019  Cohort 1 | University of Southern Mississippi | | Kinesiology | BS (2015) Kinesiology  MA (2017) Kinesiology  PhD (2020) Kinesiology | Occupational Sitting with High Sensitivity C-Reactive Protein: |
| 1.4 | 2019  Cohort 1 | UMMC | | Population Health Science | MB (2008) Preventive Med  MPH (2016) Epidemiology/Biostatistics | Anger and Stroke |
| 1.5 | 2019  Cohort 1 | UMMC | | Population Health Science | BS (2014) Speech-Language Path  MS (2015) Speech- Language Pathology  PhD (2022) Population Health | Financial Stress and Allostatic Load in Women |
| 1.6 | 2019  Cohort 1 | University of Mississippi | | Health & Kinesiology | BS Ed (2017) Exercise Physiology  MS (2018) Exercise Physiology  PhD (2023) Health Behavior | Leisure-time Physical Activity and Hypertension |
| 1.7 | 2019  Cohort 1 | UMMC | | Population Health Science | BS Nursing (2007)  MS Nursing & Health Care Administration (2011)  DNP Nursing Practice (2022) | NA |
| 1.8 | 2019  Cohort 1 | UMMC | | Population Health Science | BS Business Administration (2007)  MBA Business Administration (2011) | NA |
| 2.1 | 2020  Cohort 2 | University of Mississippi | | Pharmacy | AA (2018) Pre-Pharmacy  BS (2019) Pharmaceutical Sciences  PharmD (2023) | Adherence to Hypertension Medication |
| 2.2 | 2020  Cohort 2 | University of Mississippi | | Pharmaceutical Sciences/Pharmacology | BA (2019) Biology/Biological Sciences  PhD (2022) Biomolecular Sciences | C-reactive Protein and Stroke Incidence |
| 2.3 | 2020  Cohort 2 | UMMC | | Neuroscience | BS (2018) Chemistry  PhD (2023) Neuroscience | Parity and Left Ventricle Hypertrophy |
| 2.4 | 2020  Cohort 2 | Mississippi State University | | Food Science, Nutrition & Health | BS (2015) Nutrition  MS (2019) Nutrition  PhD (2022) Nutrition | Healthy Eating Index and Metabolic Syndrome |
| 2.5 | 2020  Cohort 2 | UMMC | | Medicine/PhD Pharmacology & Toxicology | BA (2015) Chemistry  MD (2022) Medicine  PhD (2022) Experimental Therapeutics and Pharmacology | Vitamin D and Type II Diabetes |
| 2.6 | 2020  Cohort 2 | University of Southern Mississippi | | Counseling Psychology | BS (2010) Social Work  MA (2022) Psychology  PhD (2024) Counseling Psychology | Social Support and Tobacco Smoking |
| 2.7 | 2020  Cohort 2 | UMMC | | Medicine | BS (2020) Biology/Pre-Medicine  MD (2024) Medicine | NA |
| 3.1 | 2021  Cohort 3 | University of Mississippi | | Pharmacy Administration | BS (2016) Pharmaceutical Sciences  PharmD (2020) Pharmacy  MS (2022)  PhD (2025) Pharmacy Administration | Access to Health Care and Heart Failure |
| 3.2 | 2021  Cohort 3 | UMMC | | Medicine | BS (2020) Biological Sciences  MD (2024) Medicine | Age of Menarche and CVD |
| 3.3 | 2021  Cohort 3 | University of Mississippi | | Social Welfare | BA (2011) Psychology in Social Work  MA (2014) Psychology & Education  MA (2017) Anthropology & History  MSW (2019) Social Work | Sleep and Diabetes |
| 3.4 | 2021  Cohort 3 | University of Mississippi | | Pharmacy Administration | BS (2016) Pharmaceutical Sciences  PharmD (2020) Pharmacy  PhD (2024) Pharmacy Administration | Use of Complementary Medicine |
| 4.1 | 2022  Cohort 4 | University of Mississippi | | Pharmacology | BS (2020) Psychology/Biology | Metabolic Syndrome and C-reactive Protein |
| 4.2 | 2022  Cohort 4 | Mississippi State University | | Kinesiology | BS (2017) Movement Science  MS (2020) Neuroscience | Tinnitus and Physical Activity |
| 4.3 | 2022  Cohort 4 | Mississippi State University | | Exercise Science | BS (2020) Exercise Science  MS (2022) Applied Exercise Physiology | Perceived Stress and Self-rated Health |
| 4.4 | 2022  Cohort 4 | Mississippi State University | | Kinesiology | BS (2019) Kinesiology  MS (2019) Exercise Science  PhD (2024) Exercise Science | Physical Activity and Lung Function |
| 4.5 | 2022  Cohort 4 | UMMC | | Cellular & Molecular Biology | AA (2013) General Studies  BS (2016) Biology  MS (2018) Biological Sciences  PhD (2024) Cell and Molecular Biology | Genetic Risk Scores for Left Ventricular Hypertrophy |
| 4.6 | 2022  Cohort 4 | University of Mississippi | | Health & Kinesiology | BS (2018) Exercise Science  MS (2021) Applied Exercise Physiology | Physical Activity and Arterial Stiffness |
| 5.1 | 2023  Cohort 5 | University of Mississippi | | Pharmaceutical Sciences-Pharmacy Administration | BA (2014) Chemistry  BS (2014) Biochemistry  MS (2015) Medical Health Sciences  PharmD (2021) | Cigarette Smoking and Medication Adherence |
| 5.2 | 2023  Cohort 5 | UMMC | | Population Health | BA (2016) Interdisciplinary Studies  PsyM (2020) Developmental Psychology | Resilience and Prevention of Hypertension |
| 5.3 | 2023  Cohort 5 | Mississippi State University | | Exercise Science | BS (2019) Exercise Science  MS (2022) Exercise Science | Subjective Social Status and Diet Quality by Occupation |
| 5.4 | 2023  Cohort 5 | University of Mississippi | | Physical Chemistry | BS (2016) Biochemistry  MS (2020) Biological Engineering | NA |
| 5.5 | 2023  Cohort 5 | Mississippi State University | | Food Science, Nutrition and Health Promotion | BA (1997) Sociology  MS (1999) Counselor Education Rehabilitation | Internalized and Expressed Anger and Restless Sleep |
| 5.6 | 2023  Cohort 5 | UMMC | | Medicine | BS (2018) Anthropology and Human Biology  MS (2021) Biomedical Sciences | Physical Activity and Sedentary Behavior in Waist Circumference and Heart Failure Association |
| 5.7 | 2023  Cohort 5 | University of Mississippi | | Pharmaceutical Science | BSc (2021) Chemistry | Use of Hypertensive Medications and Lung Function |
| 6.1 | 2024  Cohort 6 | University of Mississippi | | Biomolecular Sciences | BS (na) Pharmaceutical Sciences  MS (na) Pharmaceutical Sciences/  Pharmacognosy | NA |
| 6.2 | 2024  Cohort 6 | UMMC | | Medicine | BS (2017) Biological Sciences  MS (2019) Biomedical Sciences | NA |
| 6.3 | 2024  Cohort 6 | UMMC | | Medicine | BA (2021) English Literary Studies  MS (2022) Biomedical Sciences | NA |
| 6.4 | 2024  Cohort 6 | UMMC | | Population Health Science | BS (2013) and MS (2014) Nursing | NA |
| 6.5 | 2024  Cohort 6 | UMMC | | Biostatistics & Data Science | BS (2019) Mathematics  MS (2021) Biostatistics & Data Science | NA |
| 6.6 | 2024  Cohort 6 | UMMC | | Medicine | BS (2020) Biology | NA |

NA: Not available
